# Efficacy of Supratherapeutic Duloxetine Combined With Cognitive-Behavioral Therapy in Severe Treatment-Resistant Obsessive-Compulsive Disorder With Comorbid Depression: A Case Report

**DOI:** 10.7759/cureus.74541

**Published:** 2024-11-26

**Authors:** Syed Ali Bokhari, Mohamed H Nasr, Yasmeen T Alawadhi

**Affiliations:** 1 Psychiatry, Al Amal Psychiatric Hospital, Emirates Health Services, Dubai, ARE; 2 Pharmacy, Al Amal Psychiatric Hospital, Emirates Health Services, Dubai, ARE; 3 Psychology, Maudsley Health, Al Amal Psychiatric Hospital, Dubai, ARE

**Keywords:** cognitive behavioural therapy (cbt), duloxetine, obsessive-compulsive disorder (ocd), psychiatry, psychology, treatment-resistant ocd, yale-brown obsessive-compulsive scale

## Abstract

This case report discusses the treatment of a 42-year-old male with over a decade of treatment-resistant obsessive-compulsive disorder (OCD) and comorbid major depressive disorder (MDD). The patient underwent various pharmacological and psychotherapeutic treatments, including multiple antidepressants and cognitive-behavioral therapy (CBT), yet experienced only partial symptom relief. At baseline, the patient’s depressive symptoms were severe, with a Hamilton Depression Rating Scale (HAM-D) score of 28, and his obsessive-compulsive symptoms were marked, with a Yale-Brown Obsessive-Compulsive Scale (Y-BOCS) score of 34. Due to the chronicity and severity of symptoms, his treatment plan was adjusted to include a high dose of duloxetine, a serotonin-norepinephrine reuptake inhibitor (SNRI), which was gradually titrated to 180 mg/day with close monitoring. Within weeks of this dose adjustment, the patient experienced significant improvements in both depressive and obsessive-compulsive symptoms. The patient sustained these improvements over six months, reporting enhanced functional capacity and quality of life with minimal side effects, with his HAM-D and Y-BOCS scores reduced to 8 and 12, respectively. This case highlights the potential benefits of high-dose duloxetine in complex, treatment-resistant cases and underscores the value of combining pharmacotherapy with CBT to achieve durable outcomes. The report adds to emerging evidence suggesting that supratherapeutic dosing of SNRIs may be a valuable option for patients unresponsive to standard treatments. Further research is encouraged to evaluate the broader applicability and safety of this approach.

## Introduction

Duloxetine, a serotonin-norepinephrine reuptake inhibitor (SNRI), is primarily approved for the treatment of major depressive disorder (MDD) and generalized anxiety disorder (GAD). As a thiophenepropylamine derivative, duloxetine has a distinct chemical structure that facilitates its dual-action mechanism on serotonergic and noradrenergic pathways [1,2].

Emerging evidence suggests its potential utility in obsessive-compulsive disorder (OCD), particularly in treatment-resistant cases. Selective serotonin reuptake inhibitors (SSRIs) remain the first-line pharmacotherapy for OCD; however, approximately 30% of patients fail to achieve significant symptom relief, underscoring the need for alternative strategies. SNRIs such as duloxetine, with their dual action on serotonergic and noradrenergic pathways, have been proposed as promising options for patients unresponsive to standard therapies [3,4].

Although standard dosing regimens of duloxetine, between 30 and 120 mg/day, are effective for many for MDD or GAD, some cases of treatment-resistant MDD and OCD may require supratherapeutic doses to achieve meaningful clinical improvements. Limited but compelling reports have highlighted symptom alleviation in individuals receiving higher-than-typical doses of duloxetine [4-7].

This case report explores the potential of high-dose duloxetine in addressing severe treatment-resistant OCD with comorbid MDD, offering insights into its tolerability and efficacy when combined with cognitive-behavioral therapy (CBT) to support sustained symptom management.

## Case presentation

Mr. X, a 42-year-old Middle-Eastern male, presented with a chronic history of MDD and OCD, with symptoms persisting for over 10 years despite multiple interventions. His depressive symptoms included pervasive low mood, lack of energy, persistent feelings of hopelessness, and anhedonia, all of which severely impacted his motivation and functionality. Additionally, his OCD symptoms were characterized by health-related obsessions, primarily a fear of contamination, and compulsive behaviors such as handwashing and checking. These compulsive behaviors disrupted his daily life, rendering him unable to maintain consistent employment or engage meaningfully in social and familial relationships, leading to isolation and conflict within his family. There was no history of trauma, substance use, or significant past medical conditions.

At his initial presentation to our outpatient clinic in 2021, Mr. X exhibited an anxious affect, and intrusive thoughts, but preserved insight. Baseline investigations, including laboratory tests and vital signs, were unremarkable. His Yale-Brown Obsessive-Compulsive Scale (Y-BOCS) score was 34, reflecting severe obsessive-compulsive symptoms. On the obsession subscale (17/20), his intrusive thoughts occupied over eight hours daily (4/4), caused extreme functional interference (4/4), and led to marked distress (4/4). Despite occasional attempts to resist (2/4), he had minimal control over these thoughts (3/4). On the compulsion subscale (17/20), ritualistic behaviors such as handwashing and checking consumed over eight hours daily (4/4), caused extreme disruption (4/4), and were accompanied by significant distress when interrupted (4/4). His resistance to compulsions was mild (2/4), and he struggled to exert control over them (3/4). His Hamilton Rating Scale for Depression (HAM-D) score was 28, reflecting severe depressive symptoms that co-occurred with his OCD. He scored 4/4 for depressed mood, 3/4 for feelings of guilt, and 1/4 for passive thoughts about death. Sleep disturbances included difficulty falling asleep (2/4), frequent awakenings (3/4), and early morning awakening (2/4). Functional impairment was severe, with a score of 4/4 for work and activities, and psychomotor retardation was evident (3/4), though no agitation was observed (0/4). Anxiety symptoms were marked, with psychological manifestations such as persistent worry, apprehension, and difficulty concentrating (3/4), and somatic manifestations such as physical tension, palpitations, and generalized bodily aches (2/4). Mild appetite loss (1/4), fatigue, and persistent physical exhaustion were also reported.

At that time, his treatment regimen consisted of duloxetine 60 mg once daily, risperidone 2 mg at bedtime, amitriptyline 25 mg at bedtime, and propranolol 10 mg twice daily. His medication history revealed trials of multiple selective serotonin reuptake inhibitors (SSRIs), including fluoxetine (60 mg/day), paroxetine (50 mg/day), sertraline (200 mg/day), and the serotonin-norepinephrine reuptake inhibitor (SNRI) venlafaxine (225 mg/day). Each of these medications had been administered at adequate therapeutic doses and durations (six to eight weeks) but failed to achieve significant symptom relief, with frequent relapses. This history of partial or inadequate response to multiple pharmacological interventions necessitated a reassessment of his treatment plan.

In late 2021, Mr. X reported independently reducing the dose of duloxetine to 30 mg daily, hoping to minimize medication use. However, this led to a significant worsening of his obsessive thoughts and compulsive behaviors. His Y-BOCS and HAM-D scores were 32 and 26, respectively, reflecting severe symptomatology. The treating psychiatrist restored the duloxetine dose to 60 mg daily. While some stabilization was observed, his symptoms remained insufficiently controlled, with persistent intrusive thoughts and compulsions disrupting his daily functioning.

Approximately four weeks after returning to 60 mg/day of duloxetine, the dose was increased to 60 mg twice a day (120 mg/day). At this dose, moderate improvement was noted, with fewer episodes of intense sadness and a slight increase in daily energy levels. His HAM-D score improved to 20, and his Y-BOCS score decreased to 23. The duration of his compulsive rituals, particularly checking behaviors, reduced from approximately six to eight hours daily to four to five hours daily. However, compulsive checking rituals and intrusive thoughts persisted, though somewhat attenuated, highlighting the need for further optimization of his treatment plan.

In August 2022, following a comprehensive review of his treatment plan, duloxetine was titrated to 60 mg thrice a day (180 mg/day), a supratherapeutic dose. This decision was based on the potential for duloxetine, an SNRI, to modulate both serotonin and norepinephrine neurotransmission at higher doses, targeting the dual symptoms of MDD and OCD more effectively. Within a month, Mr. X reported significant improvements in both depressive and obsessive-compulsive symptoms. His HAM-D score improved to 14, and his Y-BOCS score dropped to 15. The duration of his compulsive checking rituals occurred 20-25 times daily, with intrusive thoughts reducing to three to four hours of his day. He described feeling more hopeful about the future, with consistent energy levels and reduced compulsive urges, allowing him to regain control over his actions for the first time in over a decade. These improvements greatly reduced his anxiety and distress.

Between September 2023 and March 2024, Mr. X underwent 13 sessions of CBT with a specific focus on exposure and response prevention (ERP). These sessions were scheduled every two to four weeks over a six-month period and were tailored to his progress and treatment needs. The ERP-focused therapy targeted his compulsions by systematically exposing him to feared stimuli while preventing ritualistic responses, aiming to help him directly confront his intrusive thoughts and reduce avoidance patterns. These symptoms severely disrupted his ability to work, maintain social relationships, and manage daily responsibilities. His Y-BOCS and HAM-D scores prior to initiating ERP were 13 and 12, respectively, reflecting continued improvement from the pharmacological intervention concurrent with the CBT.

During the course of therapy, Mr. X demonstrated notable improvements. By the end of the treatment, the frequency of checking rituals had reduced to two to three times daily, and the intensity of intrusive thoughts was markedly diminished. He described being able to redirect his focus more effectively and regain control over compulsive urges, which significantly alleviated his functional impairments. His Y-BOCS score improved to 11, reflecting a substantial reduction in symptom severity, while his HAM-D score dropped to 10, indicating significant alleviation of depressive symptoms. This structured psychotherapeutic approach complemented his pharmacological management, enabling him to consolidate therapeutic gains and achieve better functionality.

The structured psychotherapeutic approach complemented pharmacotherapy, enabling Mr. X to engage more fully in ERP exercises as his symptom burden decreased with duloxetine at 180 mg/day. He reported being able to complete daily tasks without overwhelming compulsions or debilitating intrusive thoughts, marking a transformative improvement in his functionality. His relationships and social engagement also improved, as he began participating more actively in family interactions and social settings, contributing to a restored sense of normalcy and autonomy. He achieved a state of functional stability that he described as life-changing.

**Figure 1:**
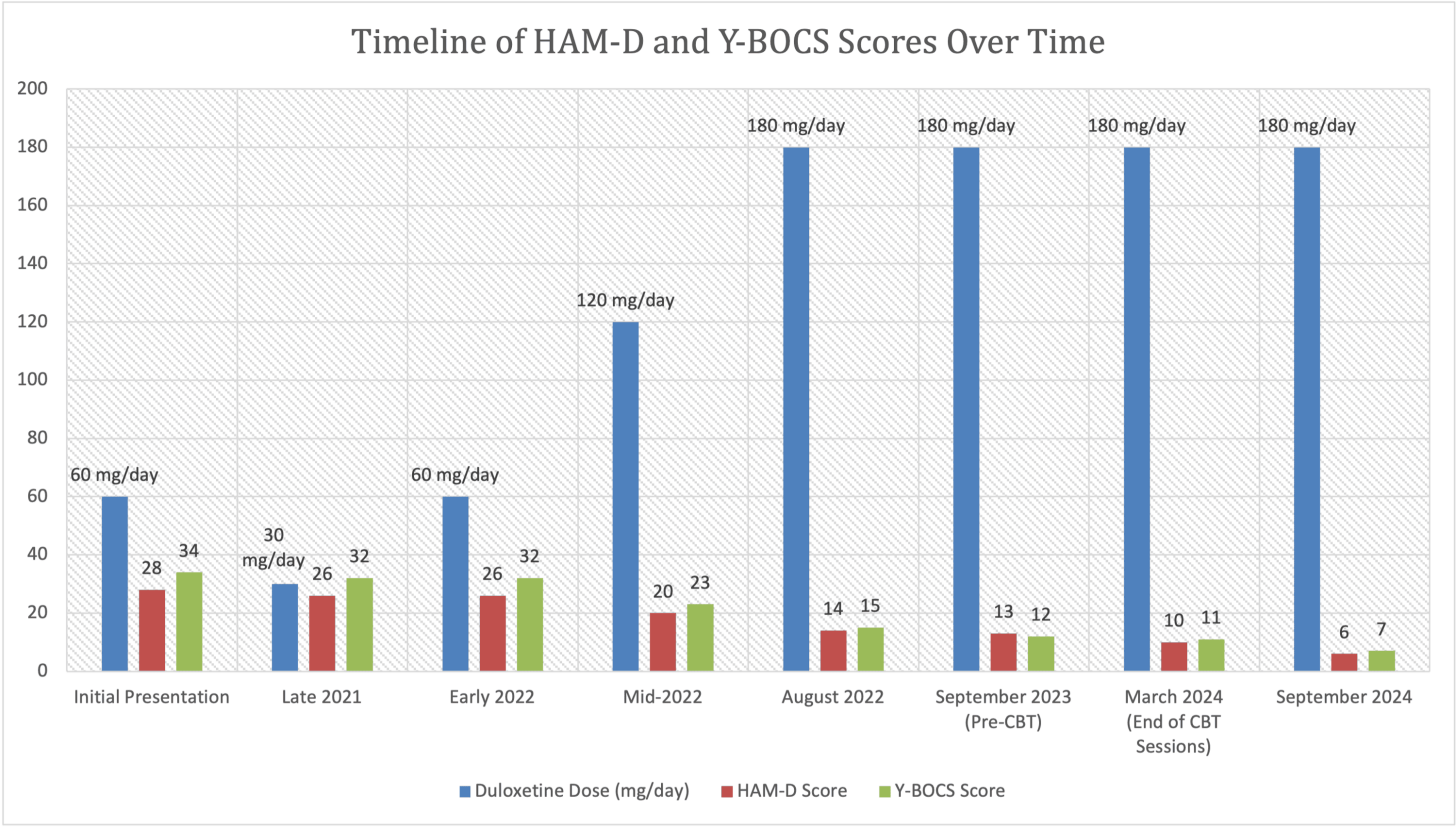
Trends in the Hamilton Depression Rating Scale (HAM-D) and Yale-Brown Obsessive Compulsive Scale (Y-BOCS) Scores Over Time, with Corresponding Duloxetine Doses.

Mr. X had achieved sustained symptom remission. Six months post-completion of the CBT sessions, his HAM-D and Y-BOCS scores were 6 and 7, respectively (Figure 1). He continues on duloxetine 60 mg thrice daily (180 mg/day), maintaining his remission even after the conclusion of CBT sessions. He expressed satisfaction with his treatment regimen and demonstrated confidence in managing residual OCD symptoms using cognitive tools acquired during therapy. His experience highlights the potential utility of high-dose duloxetine in complex, treatment-resistant cases of MDD and OCD, particularly when integrated with ERP-focused CBT to support long-term symptom management, functional recovery, and achieving meaningful and durable outcomes in treatment-resistant cases of MDD and OCD.

## Discussion

This case illustrates the potential benefits of supratherapeutic dosing of duloxetine in managing treatment-resistant MDD with comorbid OCD. Given that OCD is often unresponsive to first-line SSRIs, as seen in approximately 30% of cases, the need for alternative treatment approaches is evident [3]. While SSRIs are typically preferred, SNRIs such as duloxetine may offer additional benefits by modulating both serotonin and norepinephrine systems, addressing both depressive and obsessive-compulsive symptoms simultaneously.

The dual-action mechanism of duloxetine increases extracellular serotonin and norepinephrine levels, improving core depressive symptoms such as anhedonia and intrusive thoughts while enhancing energy and stress regulation. Unlike SSRIs, which act primarily on serotonin, SNRIs also modulate norepinephrine, which may play a critical role in alleviating compulsive behaviors and addressing dual symptom clusters often seen in patients with comorbid OCD and MDD. Duloxetine’s specificity for serotonergic and noradrenergic transporters, along with its negligible affinity for muscarinic, histaminergic, and cholinergic receptors, ensures a favorable side effect profile compared to tricyclic antidepressants, thus improving treatment adherence [1,8,9]. It has been observed that dual-action agents might offer better results in OCD patients with comorbid depression due to the combined impact on two neurotransmitter systems [4].

The case also underscores the unique challenges of treating OCD, as research shows that OCD patients tend to exhibit lower responses to both antidepressants and placebo compared to other anxiety disorders [10]. This reduced responsiveness likely contributes to the high rates of treatment resistance in OCD, suggesting a need for innovative strategies, such as supratherapeutic dosing, to achieve clinically meaningful improvements. In this context, high-dose duloxetine may offer a viable option for patients with refractory symptoms who do not achieve adequate relief from standard-dose SSRIs or SNRIs [4,7,8].

Mr. X’s response to high-dose duloxetine at 180 mg/day aligns with findings in the literature suggesting that supratherapeutic dosing may yield enhanced symptom control in treatment-resistant cases. Studies have highlighted that doses exceeding the typical maximum for duloxetine can provide significant symptom alleviation, even in patients unresponsive to conventional therapies [4-7]. For example, a similar case of treatment-resistant OCD and MDD achieved full remission within 12 weeks of initiating duloxetine at 180 mg/day, with Y-BOCS and HAM-D scores of 6 and 4, respectively [6,11,12]. This aligns with Mr. X’s substantial improvement, as reflected by reductions in his Y-BOCS and HAM-D scores, further supporting the feasibility of high-dose duloxetine in addressing persistent symptoms.

The tolerability of high-dose duloxetine is another critical factor. Although increased doses are associated with higher risks of side effects, Mr. X’s minimal adverse effects suggest that careful monitoring can ensure safety. This finding is consistent with studies showing that, while gastrointestinal or sleep disturbances may occur at higher doses, they tend to resolve over time with appropriate management [13]. Such evidence underscores the importance of individualized treatment strategies and close supervision when using supratherapeutic dosing.

The integration of CBT further highlights the value of combining pharmacotherapy with evidence-based psychotherapies. CBT, particularly exposure and response prevention, is a well-established treatment for OCD and likely reinforced the therapeutic effects of duloxetine in Mr. X’s case. By equipping him with tools to confront intrusive thoughts and manage compulsive behaviors, CBT promoted sustainable symptom management and enhanced overall outcomes. This combination underscores findings in the literature emphasizing the importance of holistic approaches for severe OCD [3,6,7].

Despite its promise, the broader applicability of high-dose duloxetine remains uncertain due to the limited evidence currently available. One study suggested that duloxetine could be as effective as other agents such as sertraline [14]. While this case underscores its potential efficacy, further research is required to validate its use across diverse patient populations. Studies focusing on optimal dosing strategies, patient selection criteria, and long-term safety profiles are critical to establishing high-dose duloxetine as a standardized treatment option for refractory OCD and MDD [5,7].

This case highlights the potential of high-dose duloxetine as a viable therapeutic option for individuals with treatment-resistant OCD and MDD. The patient’s sustained improvement at 180 mg/day, with minimal side effects, emphasizes the value of personalized and flexible treatment approaches that combine pharmacological and therapeutic interventions. However, the limited data on long-term safety and efficacy necessitate careful monitoring when using supratherapeutic doses. Larger clinical trials are essential to confirm these findings and provide robust evidence to guide routine psychiatric practice. These results advocate for cautious clinical decision-making and ongoing research to establish the broader safety, efficacy, and applicability of high-dose duloxetine.

## Conclusions

This case highlights the efficacy of high-dose duloxetine combined with CBT in managing treatment-resistant OCD and comorbid treatment-resistant MDD. The patient’s sustained symptom remission with duloxetine at 180 mg/day, with minimal side effects, suggests that carefully monitored supratherapeutic dosing can be a viable option in complex cases unresponsive to conventional treatments. While promising, these findings underscore the need for further research to guide clinical decision-making and optimize treatment strategies for individuals with severe and treatment-resistant psychiatric conditions.
